# Looking for predictive factors of clinical response to adsorptive granulocyte and monocyte apheresis in patients with ulcerative colitis: markers of response to GMA

**DOI:** 10.1186/1471-230X-13-27

**Published:** 2013-02-12

**Authors:** Yoko Yokoyama, Mikio Kawai, Ken Fukunaga, Koji Kamikozuru, Kazuko Nagase, Koji Nogami, Tomoaki Kono, Yoshio Ohda, Masaki Iimuro, Nobuyuki Hida, Shiro Nakamura, Hiroto Miwa, Takayuki Matsumoto

**Affiliations:** 1 Division of Lower Gastroenterology, Department of Internal Medicine, Hyogo College of Medicine, 1-1 Mukogawa, Nishinomiya, 663-8501, Hyogo, Japan; 2 Division of Upper Gastroenterology, Department of Internal Medicine, Hyogo College of Medicine, Hyogo, Japan

**Keywords:** Ulcerative colitis, Predictive factors of clinical response, Duration of ulcerative colitis, Multiple logistic regression analysis, Granulocyte and monocyte adsorptive apheresis, Receiver operating characteristic, Univariate analyses

## Abstract

**Background:**

Adsorptive granulocyte and monocyte apheresis (GMA) with an Adacolumn in patients with ulcerative colitis (UC) has been applied as a non-pharmacological treatment strategy, but the efficacy has been encouraging as well as discouraging, depending on patients’ demography at entry. In this study, we looked for predictive factors for clinical response to GMA in patients with UC.

**Methods:**

In a retrospective setting, 43 outpatients who had been treated with GMA for active UC were evaluated. Patients were divided into remission group and non-remission group based on Lichtiger’s clinical activity index (CAI) before and after 10, once a week GMA sessions. The efficacy was analysed in relation to patients’ demographic variables. To determine predictive factors that closely related to the response to GMA, receiver operating characteristic (ROC) curve, and multiple logistic regression analyses were applied.

**Results:**

After 10 GMA sessions, the overall clinical remission rate (CAI < 4) was 53.5%. Multiple logistic regression and ROC analyses showed that the interval between relapse and the first GMA session was a significant and independent predictive factor for clinical response to GMA (P = 0.016); the clinical response was better in patients who received GMA immediately after a relapse and vice versa. Likewise, univariate analyses showed that, the duration of UC (P = 0.036) and the cumulative prednisolone (PSL) dose (P = 0.006) before the first GMA session were significantly greater in the GMA non-responder group as compared with the responder group. Additionally, a lower white blood cell (WBC) count at first GMA session was related to clinical response to GMA (P = 0.032).

**Conclusions:**

In this study, patients with a short duration of UC and low cumulative PSL dose seemed to respond well to GMA. However, we found that the best responders were patients who received GMA immediately after a clinical relapse. Additionally, GMA was effective in patients with low WBC count at the first GMA session. The findings of this study should spare medical cost and reduce morbidity time for many patients, relevant for decision making in clinical settings.

## Background

Ulcerative colitis (UC) is one of the two major forms of the idiopathic inflammatory bowel disease (IBD), which afflicts millions of individuals throughout the world with symptoms that impair performance and quality of life [1,2]. Although the aetiology of UC is still not fully understood, elevated and activated myeloid lineage leucocytes (granulocytes and monocytes) potentially are significant factors in the exacerbation and the perpetuation of IBD [1,3-6] by releasing inflammatory cytokines [6,7]. Accordingly, in recent years, selective depletion of myeloid lineage leucocytes by adsorptive granulocyte and monocyte apheresis (GMA) with an Adacolumn has been applied as a non-pharmacologic treatment strategy in patients with UC [6-15]. However, the efficacy outcomes have been promising [6-14] as well as disappointing [15], with evidence that demographic variables of the patients at entry might indicate response to GMA [16]. In line with this assertion, even in Japan where GMA is most widely used, the popular opinion is that patients’ disease activity level, location and duration of disease, the extent of mucosal damage, hitherto response to conventional medications, and other known causes of refractoriness like cytomegalovirus infection should be taken into consideration to select patients for GMA therapy [16-18].

However, until now, it has been difficult to select the right patients for therapeutic GMA due to lack of established biomarkers of clinical response to this non-pharmacologic treatment intervention. The routinely applied weekly GMA is thought to be inadequate for efficient depletion of activated myeloid lineage leucocytes in patients with severe disease. Based on this perception, Sakuraba, et al. [6] have reported that GMA at 2 sessions per week was better than one session per week both with respect to efficacy rate and time to the disappearance of UC symptoms. Likewise, Yoshimura, et al. [19] increased the processed blood volume per session and reported significantly better efficacy rate. Therefore, there have been various attempts to improve the cost effectiveness of GMA. In this study, we were interested to identify markers of clinical response to GMA. Based on this intention, we looked at clinical response and patients’ demographic variables. The 43 patients we included in this study had been treated with GMA, and were reviewed in a retrospective setting.

## Methods

### Ethical considerations

In Japan, GMA with the Adacolumn is a Ministry of Health approved therapeutic option for patients with active IBD. Accordingly, this method is a standard therapy at our institute, where all included patients had been treated prior to this retrospective investigation by the physicians who undertook this retrospective study. However, prior to treatment, physicians ask the patients to choose either GMA or conventional pharmacologics, and at that time, patients are briefed on the nature of the procedures involved with respect to GMA. Additionally, our investigation protocol was reviewed and approved by the Ethics Committee of Hyogo Medical College Hospital (the study site). During the active treatment, adherence was made to the Principle of Good Clinical Practice at all times.

### Study design and aims

This was a retrospective single centre study conducted at the Hyogo College of Medicine in Japan. The eligible patients had been treated with GMA between April 2007 and March 2010 at this centre. In our database, we selected outpatients who were on daily low-dose prednisolone (PSL), average 17.2 mg/day including topical steroids, and could continue as concomitant medication during the GMA course. PSL administration had started well in advance of receiving GMA. The purpose of the study was to identify background factors as markers of clinical response to GMA. With this in mind, we factored patients’ demographic variables and their clinical response to GMA in our analyses. Additionally, we aimed to assess the position of GMA as an add-on or as a mono-therapy in patients with active UC, and this was one reason for selecting patients who had not achieved remission while receiving PSL.

### Patient selection and efficacy assessments

**Figure 1:**
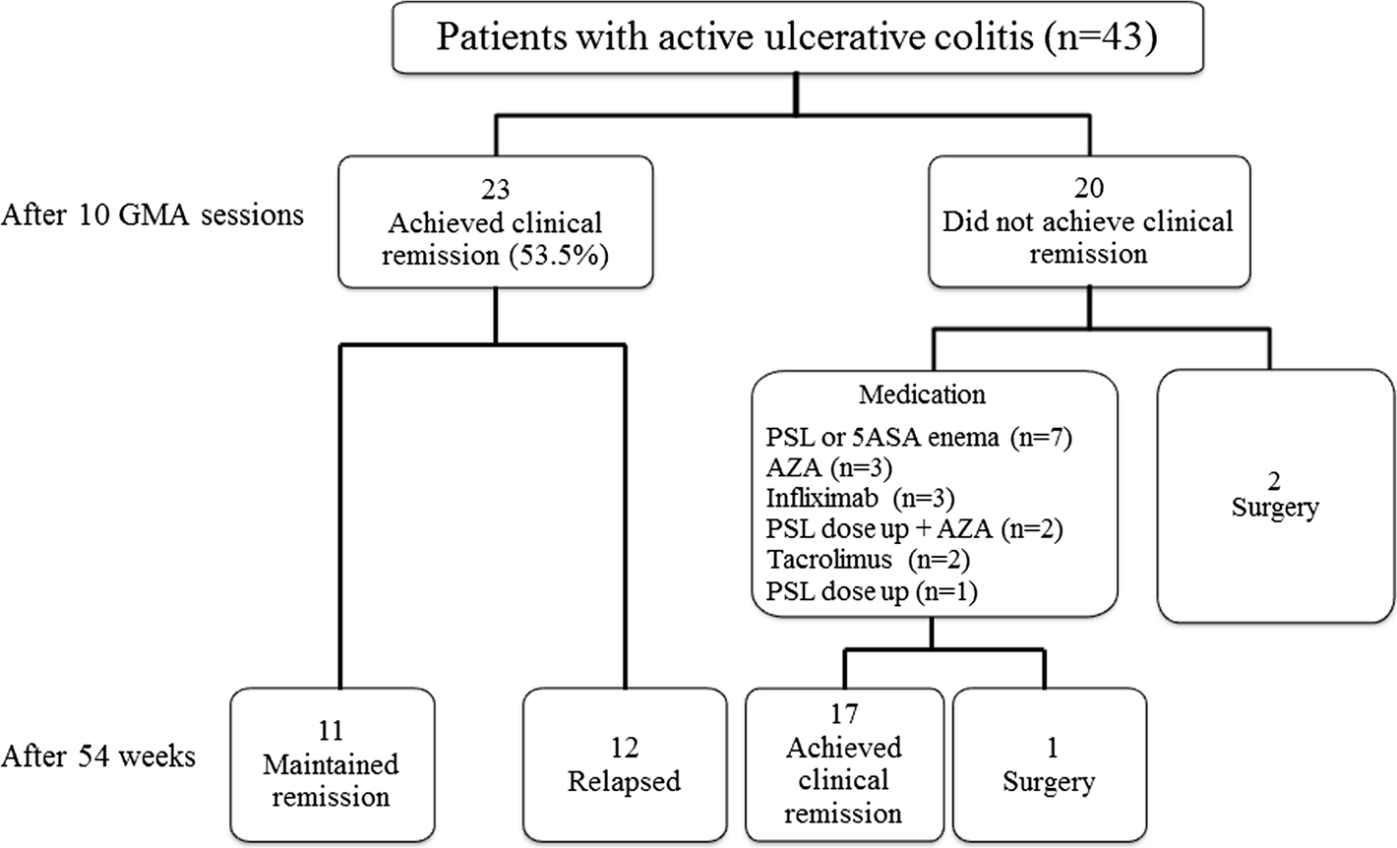
**Treatment of patients and summary of the clinical outcomes.** GMA, adsorptive granulocyte and monocyte apheresis; PSL, prednisolone; AZA, azathioprine; 5-ASA, 5-aminosalicylic acid.

Forty-three outpatients with active UC had received 10 consecutive weekly GMA, at one session per week as shown in Figure 1. Patients’ main demographic variables are presented in Table 1. Among the 43 patients, 15 were male, and 28 were female, average age 41.5 years, range 18–71 years, all with a definitive diagnosis of UC.

**Table 1:** Baseline demographic variables of the 43 eligible patients of this study

	
Gender (Female/Male)	28/15
Age (year)	41.5 ± 14.9
Duration of UC (year)	7.2 ± 7.0
CAI at 1^st^ GMA (mean ± SD)	9.1 ± 2.2
WBC at 1^st^ GMA (mean ± SD)	8900.8 ± 2436.7
CRP at 1^st^ GMA (mean ± SD)	0.7 ± 0.9
Concomitant medications	
PSL	4
AZA	1
5ASA	8
PSL + AZA	0
PSL + 5ASA	21
AZA + 5ASA	3
PSL + AZA + 5ASA	6
Extent of colitis	
Total	20
Left-sided	23

*PSL*, prednisolone; *UC*, ulcerative colitis; *CAI*, clinical activity index; *GMA*, granulocyte and monocte apheresis; *SD*, standard deviation; *5ASA*, 5-aminosalicylic acid; *AZA*, azathioprine.

Among the 43 patients, UC was steroid-refractory in 3 patients (7.0%), steroid-dependent in 25 patients (58.1%), and undetermined in 3 patients. Patients were classified as steroid-refractory or steroid-dependent according to Ogata, et al. [20]. Steroid-refractory was defined as lack of response to an oral dose of more 30 mg/day PSL over at last 2 weeks, while steroid-dependent was defined as either chronically active UC for more than 6 months or with frequent recurrence (more than once a year, or three times or more every two years) regardless of medication. Further, patients with clinical activity index (CAI) >5 were classified as having active disease. CAI ≥12, 7–11, and ≤6 were defined as severe, moderate and mild, respectively, while CAI ≤4 meant clinical remission [21]. Patients with severe, moderate and mild UC were 16.3% (7 of 43), 72.1% (31 of 43) and 11.6% (5 of 43). Among the 43 patients, 31 (72.1%) were receiving PSL as described above, and 10 (23.3%) were on azathioprine (AZA) as concomitant medication. None of the included patients had received tacrolimus, cyclosporine or infliximab prior to GMA.

Mucosal UC disease activity at entry and following a course of GMA was evaluated by using the endoscopic index (EI) described by Rachmilewitz [22]. Mucosal healing was defined as EI < 3. The 43 patients were divided into remission group and non-remission group based on CAI before and after a course of GMA. Additionally, total white blood cell (WBC) count and C-reactive protein (CRP) were determined.

### GMA procedures

GMA treatment was done with the Adacolumn (JIMRO, Takasaki, Japan) as previously described [23,24]. Briefly, the Adacolumn is filled with specially designed cellulose acetate beads of 2 mm in diameter, which serve as the column adsorptive leucocytapheresis carriers for FcγR and complement receptor bearing cells. Therefore, the carriers selectively adsorb from the blood in the column most of the granulocytes, monocytes/macrophages and a significant fraction of platelets; lymphocytes are spared and subsequently increase [6,7]. The duration of one GMA session was 60 min, at 30 ml/min. An optimum dose of sodium heparin (2000units/session) was administered during GMA as an anti-coagulant.

### Statistics

When appropriate, numerical data are presented as the mean ± SD values. Comparison of demographic variables between the remission and the non-remission groups (see above) was done by using the Mann–Whitney *U*-test or the Fisher’s exact test. Multiple logistic regression was applied to determine markers of response to GMA (Dr SSAPII for Windows). To determine predictive factors that closely related to the response to GMA, we used receiver operating characteristic (ROC) curves with area under the curve (AUC). The ROC curves for the predictive factors were plotted by using the SPSS for Windows (SPSS Inc, Chicago). The AUC was calculated, and the point with the largest AUC was defined as the point having the greatest association with the response to GMA. The best cut-off values of the predictive factors had a minimum distance from the upper left corner to the point on the ROC curve, and were distinguishable between the remission and the non-remission groups. A P value <0.05 was considered statistically significant.

## Results

### The clinical outcomes after 10 GMA sessions

**Figure 2:**
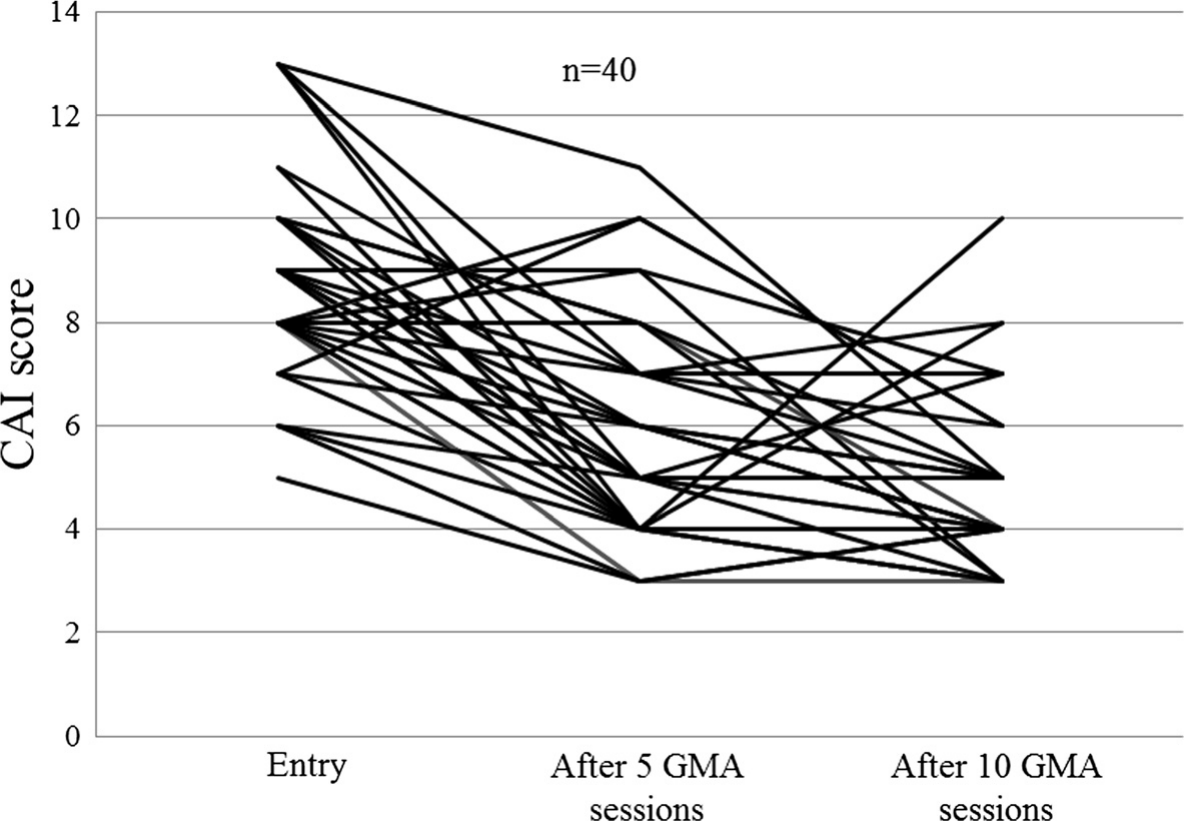
The clinical efficacy of GMA based on changes in Lichiger’s clinical activity index (CAI) evaluated at entry, after 5 and 10 GMA sessions.

**Figure 3:**
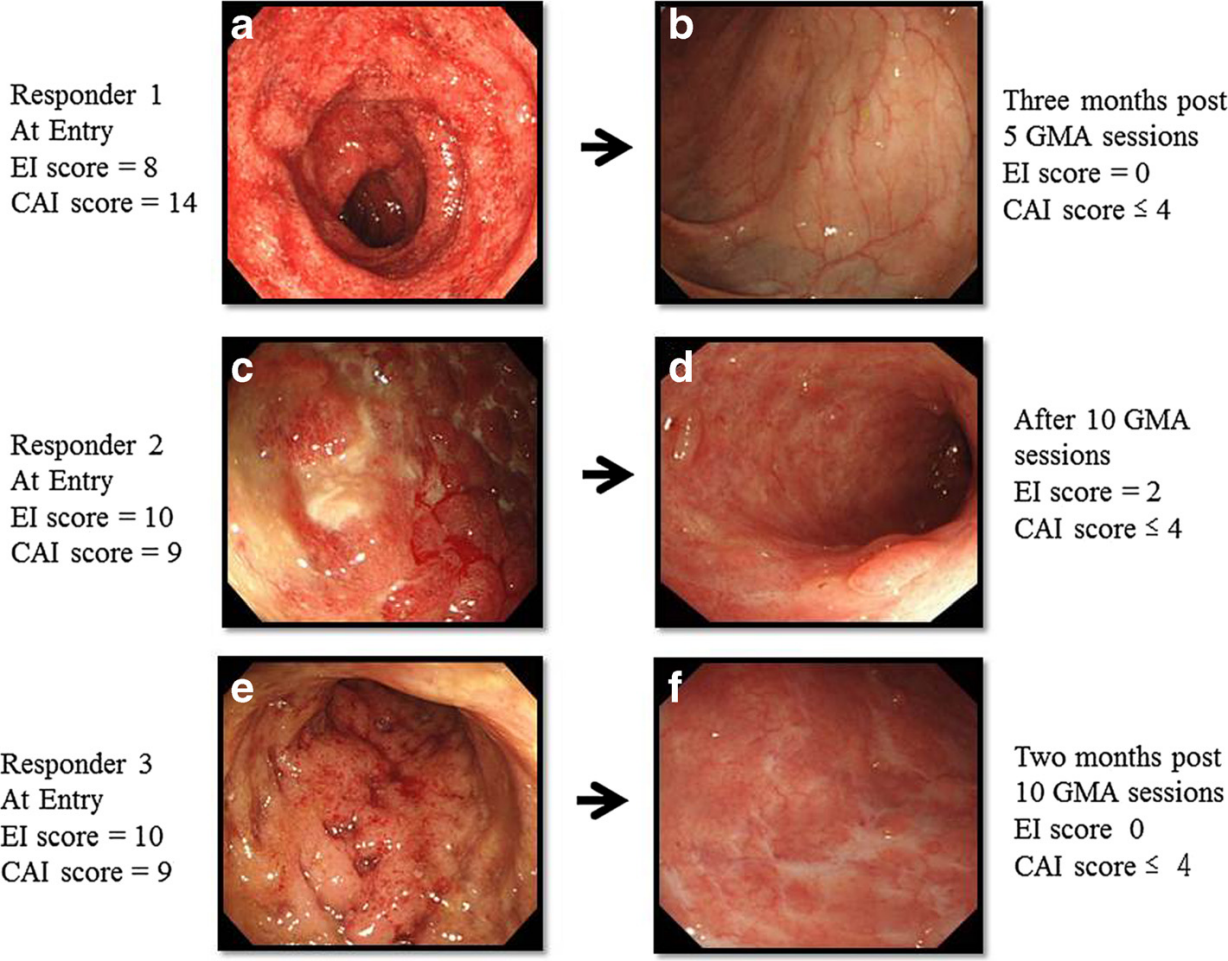
**Endoscopic findings in 3 typical responders to GMA.** All patients achieved complete remission after weekly GMA sessions. One of these patients **(Figure **3**a**) with the first UC episode had absence of vascular patterns, spontaneous bleeding, erythema, oedema and friability at entry. This patient showed an early response to GMA, after receiving just 5 GMA sessions and achieved mucosal healing (MH) a few months later **(Figure **3**b**). The second patient with steroid-dependent UC had extensive ulcers, oedematous mucosa at entry **(Figure **3**c**). This patient achieved clinical remission and partial MH after 10 GMA sessions **(Figure **3**d**). The third patient had a short duration of UC and absence of vascular patterns, spontaneous bleeding, erythema, ulcers and friability at entry **(Figure **3**e**). This patient achieved MH a few months later after 10 GMA sessions **(Figure **3**f**). These 3 patients had maintained remission at week 54 following the last GMA session.

**Figure 4:**
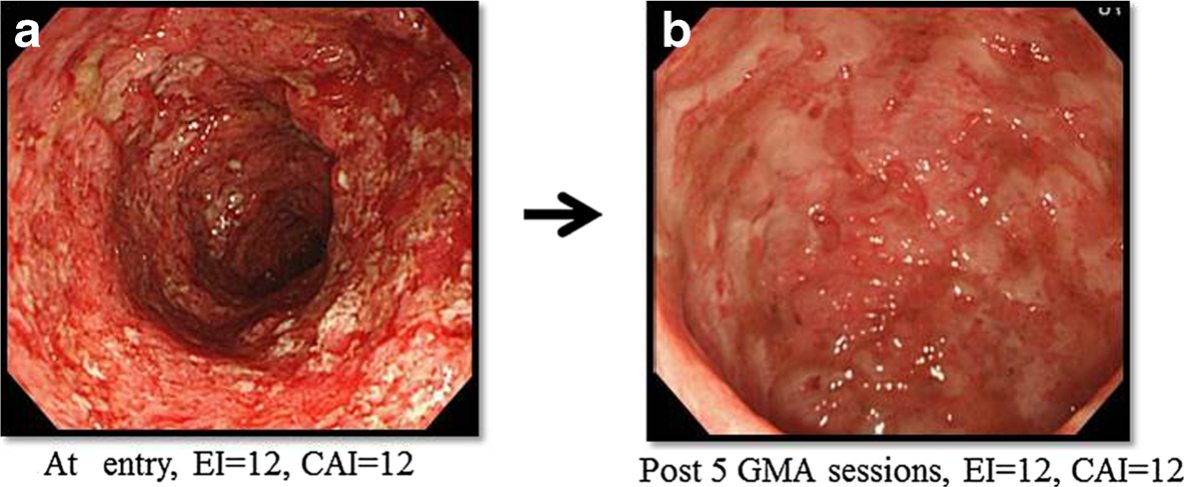
**Endoscopic findings in a typical non-responder to GMA.** At entry, this patient had extensive deep lesions and loss of mucosal tissue at UC lesion sites. GMA was discontinued after 5 sessions as it was judged futile to administer further GMA sessions to this patient.

Figure 1 shows that after 10, once a week GMA sessions, the overall remission rate was 53.5% or 23 of 43 patients. Among the 23 responders, 9 patients (39.1%) had steroid-free remission. Three of the 43 patients received ≤5 GMA sessions and therefore, 40 of 43 patients received 10 GMA sessions. The changes in the CAI score for all 40 patients who received at least 5 GMA sessions are presented in Figure 2. In the 23 responders, the average CAI score improved from 8.7 ± 1.8 at baseline to 3.8 ± 0.7 (P < 0.001). Similarly, the average EI score (n = 23) was significantly better, 8.5 ± 2.4 vs 1.8 ± 1.9, (P < 0.05), and 56.5% of those who achieved clinical remission had mucosal healing as well. The daily PSL dose was significantly tapered from 18.7 ± 14.4 mg/day at baseline to 5.2 ± 6.1 mg/day (P < 0.05) in responders after 10 GMA sessions. Colonoscopic finding in 3 typical responder patients who achieved mucosal healing are shown in Figure 3, while Figure 4 shows typical colonoscopic feature of patients who may not benefit from GMA therapy (see below).

### Predictive factors for response to GMA

We applied multiple logistic regression analysis as well as univariate analyses to identify markers of clinical response to GMA (Tables 2, and 3). In univariate analysis, there was no significant difference in age, gender, extent of colitis, CAI score, the daily PSL dose and response to steroids at baseline between the remission and the non-remission groups. The cumulative PSL dose before the first GMA session in the non-remission group was significantly higher than in the remission group, 939.2 ± 1125.9 mg vs 4578.5 ± 4752.6 mg (P = 0.006). The duration of UC was significantly shorter in the remission group as compared with the non-remission group, 6.1 ± 7.3 yr vs 8.6 ± 6.8 yr (P = 0.036). Likewise the mean interval between relapse and the first GMA session was significantly shorter in the remission group as compared with the non-remission group, 27.7 ± 31.0 days vs 76.2 ± 88.1 days (P = 0.016). Another variable, which tended to be significantly different between the remission and the non-remission groups at baseline was WBC, 8304.1 ± 2432.3/ul vs 9572.9 ± 2283.4/ul, respectively (P = 0.032). Multiple logistic regression analysis showed that the interval between relapse and the first GMA session was a significant independent predictive factor of response to GMA (P = 0.016). Therefore, GMA at an early stage following a clinical relapse is likely to induce remission of an active UC. Likewise, baseline WBC was a significant independent predictive factor for response to GMA (P = 0.025).

**Table 2:** Comparison of patients’ demographic background in the remission and the non-remission groups by univariate analysis

**Measurements**	**Remission group (n = 23)**	**Non-remission group (n = 20)**	**P-value**
Age, year	39.2 ± 13.6	44.1 ± 16.2	ns
CAI score	8.9 ± 2.1	9.3 ± 2.3	ns
Duration of UC (year)	6.1 ± 7.3	8.6 ± 6.8	0.036
Interval between relapse and the 1^st^ GMA (day)	27.7 ± 31.0	76.2 ± 88.1	0.016
PSL at the 1^st^ GMA (mg/day)	18.7 ± 14.4	17.8 ± 10.5	ns
Cumulative PSL dose before 1st GMA (mg)	939.2 ± 1125.9	4578.5 ± 4752.6	0.006
WBC at the 1st GMA (/ul)	8304.1 ± 2432.3	9572.9 ± 2283.4	0.032
Hb at the 1^st^ GMA (g/dl)	12.0 ± 1.7	12.7 ± 2.0	ns
CRP at the 1^st^ GMA (mg/dl)	0.7 ± 1.0	0.7 ± 0.8	ns

*CAI*, clinical activity index; *CRP*, C-reactive protein; *GMA*, granulocyte and monocte apheresis; *Hb*, haemoglobin; *WBC*, white blood cell; ns, not significant (p > 0.05).

**Table 3:** The outcomes of multiple logistic regression analysis for determining predictive factors of clinical response to GMA in patients with active ulcerative colitis

**Measurements**	**Beta**	**P value**	**Odds Ratio**	**95% Confidence internal**	
				**Lower**	**Upper**
Interval between relapse and 1st GMA	-0.025	0.016	0.976	0.956	0.995
WBC at 1^st^ GMA	0.000	0.025	1.000	0.999	1.000

*WBC*, white blood cell; *GMA*, granulocyte and monocte apheresis.

### The cut-off values of the 3 factors that closely related to GMA efficacy

**Figure 5:**
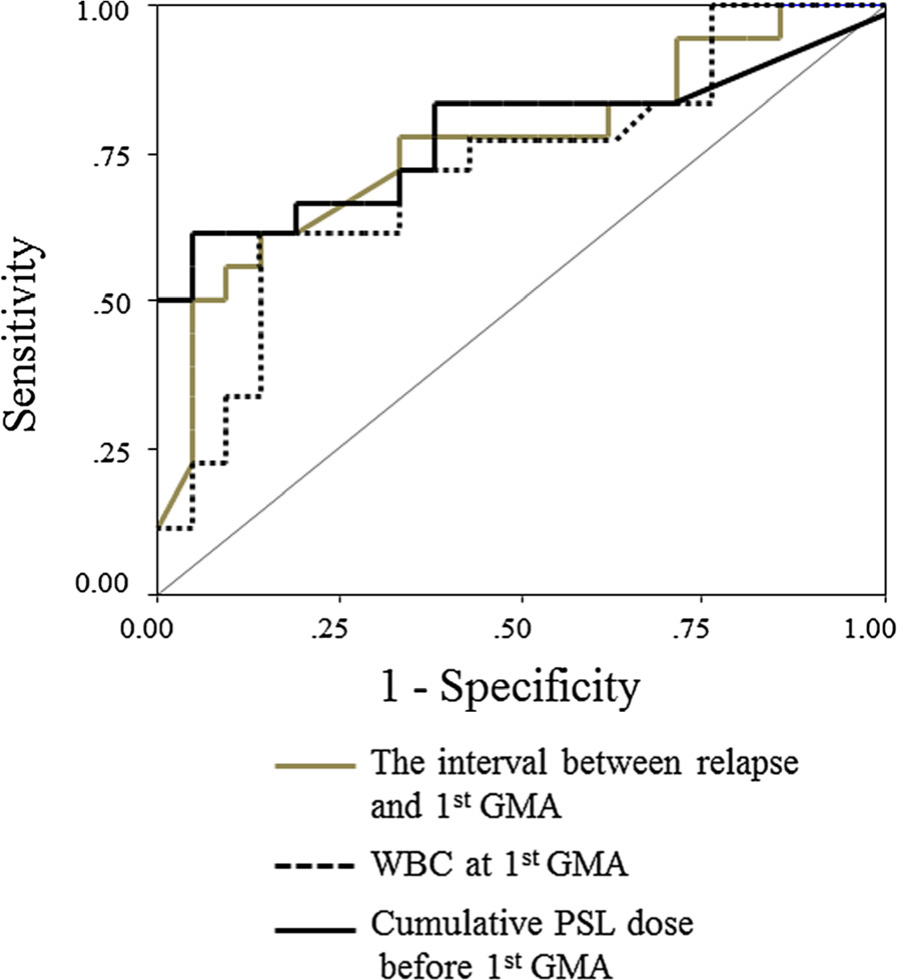
Receiver operating characteristic (ROC) curves for the interval between relapse and the first GMA, WBC at the first GMA and cumulative PSL dose before the first GMA session in patients with active ulcerative colitis.

Figure 5 shows the ROC curve for the 3 factors (the interval between relapse and the first GMA session, WBC at the first GMA session and the cumulative PSL dose before the first GMA session) in the remission group vs the non-remission group. The optimal cut-off value estimated for these factors that allows for the distinction of the remission group compared to the non-remission group was 2735.5 mg, 10160/μL and 49.5 days. The AUC of the ROC values, the cut-off values, sensitivity and specificity are summarised in Table 4. The AUC levels, sensitivity and specificity were similar to the aforementioned factors.

**Table 4:** Sensitivity, specificity, area under the curve (AUC), and confidence interval (CI) in the determination of the predictive factors of clinical response to GMA in patients with active ulcerative colitis

**Measurements**	**Cut-off value**	**Sensitivity (%)**	**Specificity (%)**	**AUC (95%CI)**
Cumulative PSL dose up to the 1^st^ GMA session	<2735.5 mg	61	95	0.78 (0.62-0.94)
WBC at 1^st^ GMA session	<10160/μL	61	85	0.72 (0.56-0.89)
Interval between relapse and 1st GMA session	<49.5 days	50	95	0.76 (0.61-0.92)

*GMA*, granulocyte and monocte apheresis; *PSL*, prednisolone; *WBC*, white blood cell.

### The prognosis of GMA responder patients

Figure 1 shows that after 54 weeks, 11 of the 23 responders had maintained remission (47.8%). In the relapse group, the average interval from the last GMA session to relapse was 4.3 months, range 1–9 months. Prior to the first GMA session (baseline), there was no obvious difference in entry demographic variables (gender, age, CAI, EI, concomitant medications, and daily PSL dose, duration) between the patients who maintained remission and those who relapsed. However in the remission group, 72.7% or 8 of 11 patients had achieved clinical remission with mucosal healing after 10 GMA sessions (complete remission).

### Study safety and patient compliance

Forty outpatients were to receive 10 GMA sessions each and all 40 patients completed their 10 weekly GMA sessions, compliance was 100%. Likewise, in this study, no serious adverse event was observed during GMA sessions. There was no other serious adverse event or opportunistic infection during our observation time, even in patients who were on PSL or AZA as concomitant medication during the course of GMA.

## Discussion

Active UC is debilitating, affects function and quality of life. The chronic nature of the disease means patients require medications throughout life. Conventional drugs often cause adverse side effects, adding to the disease complexity. It would be reasonable to assume that one major factor for physicians and patients opting for a non-pharmacological treatment intervention like GMA is to avoid adverse side effects of drugs. Ideally, physicians and in particular, patients hope treatment to be safe and effective. However, the reality of clinical practice would show that any given therapeutic intervention is effective only in a fraction of patients treated similarly for the same condition. The size of the responder fraction reflects the efficacy rate. In Japan and Europe, GMA with an Adacolumn in patients with UC has been applied as a non-pharmacological treatment strategy, but hitherto, the efficacy rate has been different, ranging from an 85% [9,12-14,25] to a statistically insignificant level [15]. Treatment failure reflects waste of resources and an increase in morbidity time for the non-responder patients. However, there is evidence that the clinical response to GMA is dictated by patients’ demographic variables at baseline [26,27] which should be evaluated. With this background in mind, in this study, we looked for predictive factors, which could guide us to select future patients who are most likely to respond to GMA.

The outcome of the present investigation might be summarized as follows. Forty-three outpatients who had received consecutive weekly GMA at one session per week, up to 10 sessions for an active UC were retrospectively reviewed. All 43 patients had active disease in spite of receiving conventional medications, the majority on PSL. At the assessment time point, the patients could be divided into remission group and non-remission group. During GMA therapy, the PSL dose was tapered and many responders became steroid free. Typically, the non-responders had extensive deep ulcers with near total loss of the mucosal tissue at the affected sites or had a long duration of UC and exposure to multiple conventional medications. Further, almost all patients who maintained their clinical remission for 54 weeks had achieved mucosal healing during the course of GMA therapy. Therefore, mucosal healing was a predictive factor for better prognosis [28-30]. Our search for predictive factors of clinical response to GMA showed that at baseline, the interval between relapse and the first GMA session was significantly shorter in the remission group as compared with the non-remission group. Additionally, the cumulative PSL dose before the first GMA session was a significant independent factor, and a high cumulative PSL dose appeared to negatively impact the efficacy of GMA. The application of logistic regression analyses indicated that GMA at an early stage following a clinical relapse was likely to induce remission of an active UC. Another significant predictive factor was total WBC at baseline. However, the percentage of granulocyte in WBC had increased in all patients (data not shown).

The publication of the first clinical experience with GMA in patients with UC by Shimoyama, et al. [31] generated significant interest in this non-pharmacological treatment intervention for patients with inflammatory diseases associated with activated myeloid lineage leucocytes [5-19,23-27,31-38]. However, as already mentioned, there is no shortage of contrasting efficacy outcome reports in the literature. In 2004, Suzuki, et al. [9] reported an 85% efficacy rate or 17 of 20 patients they treated with GMA as the first line medication. Then a controlled trial by Sands, et al. [15] reported no statistically significant difference between the sham arm and the GMA arm. The patients included in the latter study had a very different demography, had not responded to conventional medications. In contrast, all of the patients in Suzuki’s study were steroid naïve, most with a short UC duration and only needed first line medication [9,12]. Suzuki, et al.^9^ reported that the only 3 GMA non-responders in that study had defective mucosal tissue and deep ulcers. In a subsequent study, Suzuki, et al. [27] reported that all patients with a short duration of UC (about 3 months), and first episode cases readily responded to GMA, while all of the 8 GMA non-responders in that study had a very long duration of UC together with a long history of exposure to multiple conventional medications. The observations by Suzuki, et al. [9,27] were independently confirmed by Tanaka and colleagues in much larger cohorts of UC patients [16,17,25].

In the present study, the frequency of GMA was one session per week at 30 mL/min for 60 min. This is equivalent to 1800 mL of processed blood volume per GMA session. This treatment protocol might be inadequate as Sakuraba, et al. [9] found that 2 GMA sessions per week produced significantly higher efficacy rate in a shorter time as compared with one session per week. Similarly, Yoshimura, et al. [19] reported that more than 3000 mL of processed blood volume per GMA session was significantly more effective than routinely applied 1800 mL per session. Additionally, our experience suggests that unlike drug based medication, GMA demands operational skills, and maintenance of a steady blood flow.

## Conclusions

In our IBD centre, GMA is very much favoured by patients for its good safety profile. However, the clinical efficacy of GMA has been encouraging as well as disappointing depending on the baseline demographic features of the treated patients. Patients with a short duration of UC responded better and faster to GMA as compared with patients with a long duration of UC. However, we found that the best responders to GMA were patients who received GMA immediately after a relapse. Accordingly, the interval between the clinical relapse and the first GMA session was a significant predictive factor for clinical response to GMA. This indicated that GMA should be effective as a mono-therapy if applied soon following a UC episode. Additionally, patients with active UC and a long history of exposure to corticosteroids may not respond well to GMA. Other independent studies have reported that steroid naïve and patients with the first UC episode as the best responders to GMA, while patients with deep ulcers and extensive loss of the mucosal tissue at the lesion sites or patients with a long duration of UC and exposure to multiple drugs as poor responders. The bottom line might be that GMA should be applied before patients become refractory to the currently used drugs. Potentially, the information in this article should spare medical cost and reduce morbidity time for many patients, relevant for decision making in clinical settings.

## Abbreviations

AUC: Area under the curve;AZA: Azathioprine;5-ASA: 5-aminosalicylic acid;CAI: Clinical activity index;CRP: C-reactive protein;EI: Endoscopic index;FcγR: Fragment crystallizable gamma receptor;GMA: Granulocyte and monocyte adsorptive apheresis;IBD: Inflammatory bowel disease;PSL: Prednisolone;ROC: Receiver operating characteristic;UC: Ulcerative colitis;WBC: White blood cells

## Competing interests

The authors declare that they have no competing interests.

## Authors’ contributions

YY, KF, KK, KN, and TM: Conception, study design, acquisition and interpretation of the data, statistics, drafting, and preparation of the final manuscript version. MK, KN, NH, YO, MI, SN, and HM: Patient management, acquisition and interpretation of the data and review of the final manuscript draft. All authors read and approved the final manuscript.

## Authors’ information

Yoko Yokoyama and Mikio Kawai contributed to this article equally as the first co-authors.

## Pre-publication history

The pre-publication history for this paper can be accessed here:

http://www.biomedcentral.com/1471-230X/13/27/prepub
